# Supplementary material for: Are carbon emissions trading and green financial instruments synergistic? -Comprehensive quantitative research based on content analysis
Source: PLoS One. 2024 Mar 7;19(3):e0298601. doi: 10.1371/journal.pone.0298601 (PMC10919601; doi:10.1371/journal.pone.0298601)
Supplement: S1 Data — (ZIP) [file pone.0298601.s001.zip › Data/地级市Z_F工作报告.zip!/╕ú╜¿/╞╬╠∩╩╨/└┤╫╘╣1⁄2╓┌║┼í╛┬φ┐╦╩2╛▌═°í┐_2008.docx]

各位代表：

现在，我代表莆田市人民政府向大会作政府工作报告，请予审议，并请市政协委员和其他列席同志提出意见。

一、湄洲湾港口城市建设又好又快向前推进

过去的一年，在中共福建省委、省政府和中共莆田市委的领导下，全市人民深入贯彻落实科学发展观，认真学习贯彻党的十七大精神，加快推进湄洲湾港口城市建设，经济社会发展取得令人振奋的成绩，圆满完成了市五届人大一次会议确定的奋斗目标。

（一）综合实力持续提升。初步核算，全市生产总值513.81亿元，增长16.5%；全社会固定资产投资240.93亿元，增长45.9%；财政总收入45.67亿元，增长22.4%，其中，地方级财政收入23.8亿元，增长28.8%；三次产业持续发展，增加值分别增长6%、20.3%、14.2%，产业结构调整为13.0∶56.1∶30.9；工业增加值253.11亿元，增长21.4%，产值超亿元工业企业总数达到149家；企业经济效益明显提高，规模工业企业经济效益指数180.90%，提高17.36个百分点；产业集聚能力增强，十大产业集群完成规模工业产值594.54亿元，增长29.6%；节能减排取得成效，初步统计，万元规模工业增加值能耗为0.58吨标准煤，下降20.5%；农林牧渔业总产值111.51亿元，增长6.5%；社会消费品零售总额172.86亿元，增长18.5%；旅游接待人数增长6.9%，收入增长20.4%；金融运行态势良好，金融机构本外币各项存款余额406.78亿元，贷款余额299.48亿元，分别比年初增长4.5%和28.9%；城镇居民人均可支配收入14351元，增长16.9%；农民人均纯收入5628元，增长14.3%；城镇登记失业率2.19%。品牌带动取得新成效，5家企业获中国名牌产品称号，2家企业获中国驰名商标称号，华丰鞋业有限公司被国家质检总局授予出口免验资格企业，“南日鲍”获国家地理标志产品保护称号。国家浆纸质检中心落户我市。

（二）项目带动成效显著。105个在建重点项目实现年度投资122.43亿元，完成年度计划的101.7%。工艺美术城、平海湾跨海供水工程、木兰溪郑坂段主体工程顺利完成，城港、荔港、仙港、沁峤路等通港大道基本贯通；福厦铁路（莆田段）、莆秀高速公路、涵港大道、福建LNG项目秀屿接收站、莆田燃气电厂、金钟水利枢纽工程、妈祖城、莆田鞋服城等项目加快建设；向莆铁路（莆田段）、金莆供水和木兰溪霞林段防洪工程开工建设；莆田核电站、湄洲湾火电厂二期、湄洲湾北岸疏港铁路、莆田机场、罗屿25万吨级散货码头等项目的前期工作顺利推进；马来西亚云顶集团投资10亿美元的太阳能薄膜电池、新加坡金鹰集团投资8.7亿美元的差别化化学纤维和高档纸、福建国航湄洲湾造修船有限公司投资59亿元的国航造修船基地、省交通控股公司投资25亿元的东吴5个大型深水泊位、省煤炭集团投资10亿元的石城和石井风电项目、国电集团投资25亿元的煤炭中转基地项目顺利签约，鞍钢集团总投资30亿元的冷轧板项目可望签约。港口建设力度加大，临港产业取得新进展，港口吞吐量达到1613万吨，增长24%。

（三）改革开放取得突破。大力推进农村综合改革，深化粮食流通体制和林权制度改革。稳步推进投资、金融、财税、科技、价格、公共服务等改革。加强国有资产监管，推进公共资源市场化配置。鼓励企业改制上市，27家企业加快改制步伐。开展第二次全国农业普查和土地调查，审批建设用地1.78万亩，保护基本农田，加强土地整理，连续8年保持耕地占补平衡。招投标工作成效显著，全年进入市招投标中心交易项目572个，交易总额53.54亿元，节约资金7.14亿元，增值资金8.2亿元。通过招拍挂出让经营性土地21宗931.5亩，收取土地出让金24.6亿元。大力清理闲置土地，其中，清理经营性闲置土地64宗，依法收回5宗1271.4亩，促其动工59宗，补缴土地出让金和闲置费5.6亿元。扩大对外开放，成功举办莆田（北京、新疆、香港）经贸活动周、第三届中国（莆田）木业投资贸易洽谈会、“三会一节”、闽东北经济协作区联席会议等活动，赴湖南、江西、台湾、东南亚等地经贸考察，加强招商引资和对外交流。全市进出口总额21.56亿美元，增长26.1%。审批利用外资项目94个，按历史可比口径，合同利用外资5.6亿美元，增长3.1%；实际利用外资5.53亿美元，增长27.4%。引进民资项目393个，总投资310.5亿元，完成投资93.3亿元，民营经济占全市经济总量60%以上。加强莆台交往，秀屿港区被批准为福建沿海与金、马、澎直接往来货运口岸，成功举办首届莆台妈祖文化周活动，湄洲与马祖首次通航。

（四）城乡面貌明显改变。修编新一轮城市总体规划，完成“十一五”城市近期建设规划、妈祖城总体规划、荔城新区规划和30个片区控制性详细规划。大力推进城乡一体化，加大城市新区建设力度，城市建成区达到48.8平方公里，城镇化水平达到46.1%（新口径），位居全省第四位。动工城建项目80个，总投资107亿元，增长3.7倍，完成年度投资24.1亿元。完成房地产投资49.61亿元，实现建筑业总产值53.37亿元。加快旧城改造，旧体育场项目用地顺利拍卖，护城河五期、鲤中、宫口等片区改造进展顺利。完善城市公用基础设施，新增城市道路67.8万平方米。实施绿化、亮化、美化工程，新增城市绿化43.64公顷，建成区绿化覆盖率达到37%，顺利通过省级园林城市验收。城市污水处理率达到65%，垃圾无害化处理率达到83.5%。实施北洋水系整治及截污工程，护砌北洋水系堤岸10.65公里，二期工程顺利开工，努力实现“河畅水清岸绿”目标。中铁集团拟投资22亿元建设妈祖城核心区基础设施，妈祖城建设初见成效。大力推进新农村建设，建成一批新型农村住宅小区，3个村、30家农户被评为全国“绿色小康村”、“绿色小康户”。527个行政村实施“家园清洁行动”，农村人居环境不断优化。湄洲岛被授予“中国生态文明建设示范基地”。建成一批农村能源、水利、道路、电力、通讯等基础设施，新增农村沼气用户5000户，新建农村水泥公路604公里，提前实现“村村通水泥公路”、“岛岛有交通码头”和“村村通客运班车”目标。

（五）社会事业全面发展。科技综合实力不断提升，荣获全国科技进步先进城市二连冠，仙游、涵江、荔城和城厢获得全国科技进步先进县（区）称号。学前教育、义务教育、普通高中教育、职业教育和高等教育取得新成效，3所职校被评为国家级重点职校。启动莆田一中妈祖城校区等建设项目。终身教育工作不断推进，老年教育取得新进展。群众性体育运动蓬勃兴起，省运会场馆抓紧建设。文化事业加快发展，莆仙大剧院建设进展顺利，一批文化精品再创佳绩，以杨金远短篇小说《官司》改编的电影《集结号》轰动国内外，《千里送》、《杀贡鹤》等莆仙戏演出获得成功，第二届《莆田市志》编撰工作顺利推进。群众性精神文明创建活动深入开展，社会文明程度不断提高。低生育水平保持稳定，人口自然增长率为6.4‰。免费婚检4944人，免费检查妇科病近万例。社会福利事业稳步发展，殡改工作扎实推进，市福利院、市慈康医院顺利迁建。村（居）委员会顺利换届，社区建设稳步推进。信访工作得到加强。社会治安综合治理成效显著，多元化调解衔接机制初步建立，严厉打击各种犯罪活动，社会治安总体满意率进一步提高。双拥共建持续深入，蝉联“全国双拥模范城”三连冠。国防动员、民兵预备役、海防、人防等工作取得新进展。人事、编制、气象、防震、消防、保密、档案等工作进一步加强。

（六）民生保障更加有力。加大改善民生工作力度，集中财力为民办大事、办实事，让人民群众共享经济社会发展成果。全市财政用于科技、教育、卫生、文化事业和社会保障支出18亿元，增长32%。免除农村义务教育阶段学生学杂费，城乡低保家庭子女义务教育实行“两免一补”，受惠学生30多万人。市急救中心、市第五医院、莆田人民医院投入使用，市疾控中心、市第一医院医技大楼和仙游县疾控中心已经竣工。新型农村合作医疗全面推开，农村居民医疗服务水平进一步提高。对4.3万户农村困难家庭实行医疗救助，为2249名贫困白内障患者免费施行复明手术。提高居民最低生活保障标准，农村低保受益群众8.6万人，城市低保受益群众1.3万人。落实库区移民后期扶持政策，发放扶持资金2656万元。城镇新增就业2.27万人，下岗再就业2200人，农村劳动力转移就业4.23万人。发放住房公积金贷款2.72亿元，惠及1.4万户家庭。1092户城市低保家庭住房困难得到解决，“侨居造福工程”新建住房120套，“残疾人安居工程”新建住房57套，“造福工程”搬迁1850人，迁移地质灾害涉险群众2631人。加强各种涉及人身健康安全产品监管，提高产品质量和食品药品安全水平。民族、宗教、老区、妇女、儿童和残疾人等工作取得新进步。

（七）政府建设不断加强。坚持依法行政，自觉接受人大及其常委会监督，依法执行人大决议，重视和支持人民政协履行政治协商、民主监督、参政议政职能，全年共办理市人大代表建议244件、市政协提案399件，办结率均为100%。加强“一办三中心”建设，深化行政审批制度改革，减少审批事项。提高危机应急处置能力，战胜风暴潮等自然灾害，果断处置登革热等突发公共卫生事件，妥善处置“10·21”特大火灾。推进政务公开，促进市民参政议政。加大监察、审计监督力度，深入推进廉政建设和反腐败斗争，深入开展商业贿赂治理、纠风和效能工作，严肃查处涉及政府机关工作人员案件，依法惩处腐败分子。

回顾去年的工作，我们在抢抓机遇中突出发展，在战胜困难中加快发展，在开拓创新中跨越发展，主要经济指标增幅位居全省前列，经济社会取得令人瞩目的新成就。我们深切体会，必须始终坚持解放思想，深化对市情认识，以思想大解放，观念大更新，认识大飞跃，推动经济大发展；必须始终不渝坚持发展第一要务，把科学发展观贯彻落实到经济社会发展各个方面，把握发展规律，转变发展方式，提高发展质量，实现又好又快发展；必须始终注意坚持改革创新，深入推进重点领域和关键环节改革，努力形成更具活力更具开放的发展环境，增强发展的生机和活力；必须始终注重运作，着力研究解决经济社会发展中的主要矛盾，统筹协调事关全局的重大问题，通过具体运作，破解难题，化解矛盾，求实求效，推动发展；必须始终不懈坚持项目带动，以项目集聚要素，落实工作，增强发展后劲；必须始终如一坚持惠民利民，以人为本，做到发展为了人民，发展依靠人民，发展成果由人民共享。

各位代表，过去一年的成绩来之不易，这是中共福建省委、省政府和中共莆田市委正确领导的结果，是全市人民智慧和力量的结晶。在这里，我代表莆田市人民政府，向为湄洲湾港口城市建设事业付出辛勤劳动的全市人民，向给予我们支持与监督的人大代表、政协委员，各民主党派、工商联和无党派人士、各人民团体、社会各界人士，向离退休老领导、老同志，向驻莆部队、武警官兵和人民警察，向热心支持莆田发展的中央和省直属单位，向所有关心支持莆田建设的港澳台同胞、海外侨胞和国际友人，向依法纳税人，致以崇高的敬意和衷心的感谢！

在肯定成绩的同时，我们也清醒地看到，政府工作同形势的要求和人民的期望仍有差距，经济社会发展中还存在不少困难和问题，具体表现为：经济总量较小，与我市地处沿海一线的位置很不相称；产业集群不够发达，高新技术产业比重不高，第三产业尤其是现代服务业发展较慢；城乡发展还不够协调，县域经济总量偏小，工业规模不大，缺乏大项目支撑，发展后劲不足；农业基础设施薄弱，抵御自然灾害能力不强；城市功能不够完善，公共基础设施配套有待加强，城市管理水平有待提高，环境脏乱差和交通不畅问题突出；社会建设还存在薄弱环节，教育、卫生、文化等社会事业还不能完全适应人民群众日益增长的需求；就业压力较大，物价上涨幅度较高，社会保障能力较弱，部分低收入群众生活还比较困难；土地、淡水、能源、生态环境等资源约束趋紧；融资难、用工难等问题还没有根本解决；政府职能和机关作风有待进一步转变，行政审批效率不高，个别政府工作人员服务意识淡薄，有的甚至违法乱纪；安全生产、防灾减灾措施在基层尚未全面落实，发生了“10·21”特大火灾等等。我们要高度重视这些问题，采取有力措施加以解决，确保今年各项工作做得更好。

二、全力推进经济社会跨越发展

2008年政府工作的总体要求是：全面贯彻党的十七大精神，高举中国特色社会主义伟大旗帜，以邓小平理论和“三个代表”重要思想为指导，深入贯彻落实科学发展观，按照市第五次党代会和市委五届四次全会的部署，围绕“四谋发展”实践主题，坚持“四个重在”实践要领，落实“四个关键”实践要求，朝着“四求先行”实践方向，实施项目带动、品牌带动、民资回归，坚持好字优先、稳中求进、又好又快，转变发展方式，深化改革开放，推进自主创新，保护生态环境，着力改善民生，促进和谐发展，努力实现速度质量效益相协调、消费投资出口相协调、人口资源环境相协调、改革发展稳定相协调，主动融入海峡西岸经济区建设大局，加快建设湄洲湾港口城市，为建设科学发展的先行区、两岸人民交流合作的先行区作出更大的贡献。

2008年经济社会发展的主要目标：确保“超百保八争十”目标，实现生产总值630亿元，增长18%以上；工业增加值315亿元，增长21%；财政总收入55亿元，增长20.4%，其中地方级财政收入29.6亿元，增长24%；社会消费品零售总额211亿元，增长22%；全社会固定资产投资额320亿元，增长32.8%；外贸进出口总额24.5亿美元，增长14%；实际利用外资6亿美元（可比口径），增长10%；城镇居民人均可支配收入实际增长10%；农民人均纯收入实际增长8%；城镇登记失业率控制在3%以内；居民消费价格指数控制在105%以内，人口自然增长率控制在7.5‰以内；单位生产总值能耗降低3%；二氧化硫排放量下降0.5%，化学需氧量排放减少0.5%。

为完成以上目标，必须抓好以下工作：

（一）统筹城乡发展，推进新农村建设

夯实农业基础。加快构建强化农业基础的长效机制，加大强农惠农力度，巩固农业基础地位。推广农业“五新”，促进农业科技创新，壮大畜牧、水产、林产、园艺和农产品加工业，加快发展无公害农产品、绿色食品和有机食品，推进农产品标准化、规模化生产。扶持90家国家、省、市级农业产业化龙头企业，创建一批国家级、省级现代农业示范园区。鼓励土地承包经营权流转，提高农业规模效益，扩大农产品出口。建设一批高产优质的粮食生产基地，完成116万亩粮食播种任务。强化动植物疫情防控监测，抓好高致病性猪蓝耳病、禽流感等重大疫病的强制免疫。提高农村金融服务水平，扶持发展农民专业合作组织和农村服务组织。加快农业基础设施建设，推进澄峰围垦垦建、病险海堤水库、“六千”水利工程建设，实施海洋防灾减灾“百千万”工程，完善农业防灾减灾体系。

促进农民增收。落实工业反哺农业、城市支持农村和多予少取放活的方针，今年财政支农资金、政府固定资产投资和土地出让收入用于农村建设的增量要明显高于上年。加大对农民直接补贴力度，增加粮食直补、良种补贴、农机具购置补贴和农资综合直补。大力支持优质农产品生产和特色农业发展，推进农产品精深加工，通过结构优化促进农业增效。大力发展乡镇企业、家庭工业和乡村旅游，增强县域经济发展活力，提高非农收入。防止农民负担反弹，合理调控重要农产品和农业生产资料价格，保障农民对集体财产的收益性，创造条件让更多农民获得财产性收入。

建设新农村。按照“生产发展、生活宽裕、乡风文明、村容整洁、管理民主”的要求，建立促进城乡一体化发展机制，努力形成城乡发展规划、产业布局、基础设施、公共服务、社会管理一体化新格局，以城带乡，城乡融合，促进城乡一体化。抓好8个省级创新型试点村和28个市级农村新区示范点建设，建成一批新型农村住宅小区。加大农村水、电、路、通讯、广电等基础设施建设力度，改善农民生产生活条件。实施饮水安全村村通工程，建设金莆供水工程，让沿海、海岛及偏僻山区农民喝上安全卫生饮用水。完善农村公路网，新建一批农村客运站（亭），方便农民出行。大力治理农村垃圾和农村面源污染，改善农村生态环境。继续实行领导和部门单位挂钩扶贫制度，促进革命老区、少数民族地区和贫困村加快发展。“造福工程”年度搬迁2000人。

（二）实施项目带动，增强发展后劲

高度重视项目前期工作。策划生成一批先进高效、节能低排、带动力强、惠及民生的大项目、好项目，储备重大项目，形成项目滚动发展格局，通过项目带动优化结构、提升质量、增创效益，促进又好又快发展。重点抓好莆田核电站、湄洲湾火电厂二期、莆永高速公路、绕城高速公路、莆田机场、湄洲岛跨海通道、罗屿25万吨级散货码头、国电集团煤炭中转基地、国家投资开发总公司兴化湾南岸开发项目、鞍钢冷轧板等重大项目的前期工作。认真研究国家部委支持海峡西岸经济区的各项优惠政策，以国家产业目录为指导，提高项目策划生成水平，高度重视引进央属、省属企业的重点项目。

扎实推进重点项目建设。持续开展“项目攻坚年”活动，着力抓好130个在建重点项目、64个预备重点项目和31个重点前期项目，进一步提高重点建设占全社会投资比重、工业项目占重点建设投资比重，提升重点建设水平。建成LNG项目接收站、莆田燃气电厂、4万吨级木材检疫除害处理专用码头等重大项目，基本建成滨海、城港、荔港、仙港、涵港、黄北、沁峤路等疏港通道；加快建设向莆铁路（莆田段）、福厦铁路（莆田段）、莆秀高速公路、福厦高速公路（莆田段）扩建工程等大通道；开工建设湄洲湾北岸疏港铁路、LNG接收站二期、国航造修船基地、金鹰集团差别化化学纤维和高档纸、省煤炭集团石城和石井风电等一批重点项目；加快推进林浆纸一体化项目、抽水蓄能电站、金钟水利枢纽、中原港务中心油库、秀屿5万吨级多用途码头和5万吨级通用码头、东吴大型深水泊位、三江口3000吨级码头和湄洲湾航道二期疏浚工程进度。

统筹港口规划建设。完成港口总体规划报批工作，整合“三湾”资源，做好湄洲湾北岸、平海湾和兴化湾南岸岸线控制性规划，推进兴化湾南岸、平海湾海洋功能区划调整。坚持走港口、产业、城市、生态四位一体、互动发展的路子，着力构建交通网、港口群、产业群和城市群，逐步将湄洲湾北岸、平海湾和兴化湾南岸打造成为东南沿海的重要交通枢纽、内陆对外开放的出海通道、新兴的临海重化产业基地和散货转运中心、对台合作的重要平台、绿色宜居的滨海新城，以临海开发带动全市持续发展。

（三）加强自主创新，提高产业竞争力

着力提高科技创新水平。围绕建设“创新型城市”目标，继续实施三大科技发展工程，创建六大科技示范基地，开展院地科技合作，引进一批重大高新技术项目，开发一批具有自主知识产权的高科技产品。实施人才强市战略，促进“民智回归”，鼓励莆籍院士、专家、学者积极参与家乡建设，吸引海内外具有较高学术水平和一定产业背景的科技领军人才来莆创业。创建一批省级和市级技术研发中心，培育一批创新型企业。在高新技术开发区和重点企业创建研究开发机构，为企业科技创新提供服务。建设市科技中心。重视科普工作。

着力实施品牌带动。要把品牌带动作为贯彻落实科学发展观、促进又好又快发展的重要抓手，增创一批中国驰名商标、中国名牌和国家免检免验产品，鼓励企业参与国家、省级和行业标准制定，抢占行业技术标准制高点，努力形成一批优势明显的产品品牌、影响广泛的服务品牌、带动力强的区域品牌、技术领先的科技品牌和涵盖各个领域的强势品牌。通过品牌带动提升发展质量，增进经济效益，增强区域综合竞争力。

着力提高工业竞争力。走新型工业化道路，提升五个经济开发区、国家级木材贸易加工示范区、工艺美术城发展水平，引进一批上下游关联度大、市场前景好、科技含量高的重大生产性项目，促进产业集聚。依托现有产业基础和比较优势，以骨干企业、名牌产品为龙头，着力培育壮大十大产业集群。发挥“中国古典家具之都”和“中国珠宝玉石特色产业基地”品牌集聚作用，积极筹建省级珠宝质检中心和省级木雕古典家具生产中心、检验检测中心，发展工艺品特色产业。加大工业投资力度，着重抓好50个重点工业项目和86个新增产值3000万元以上的新经济增长点建设。新增产值超亿元工业企业30家，新增产值超3000万元工业企业80家，新增规模以上工业企业150家。加快建设LNG冷能利用产业园、莆田光电产业园，推进太阳能薄膜电池、光纤陶瓷插芯等高新技术项目建设进度。加快建设机电工业集中区，发展机械装备业和电子信息业。

着力提高服务业水平。认真贯彻落实国家和省有关发展服务业的政策措施，大力促进服务业发展。推进大型钢铁、煤炭、木材、粮食、小商品、建材、鞋服等专业市场建设，构建物流交易集聚区。扶植一批集运输、仓储、配送、信息为一体的“铁海公”联运现代物流企业，发展金融、保险、外轮代理、仓储信息等港口服务业。鼓励发展工程咨询、认证评估、广告创意、产品设计等商务服务业，拓展法律、科技、培训和信息服务业，巩固发展批发零售、住宿餐饮、家政服务、健身娱乐等消费性服务业。改造提升商贸流通业，推广连锁经营、特许经营、电子商务等现代经营方式和新型业态。扶持为中小企业融资服务的担保公司和典当行，筹建莆田农村商业银行，拓展金融服务领域。吸引国内外大型企业集团研发、销售、采购中心、营运总部或分支机构集聚莆田总部商务区，发展总部经济。积极发展会展业，办好第三届中国（莆田）海峡工艺品博览会和第四届中国（莆田）木业投洽会。加强住房消费引导，落实进城购房农民户籍、子女就学等政策，规范房地产市场秩序，推动房地产业健康发展。围绕创建“中国优秀旅游城市”和滨海旅游胜地目标，加快旅游资源开发，打造滨海、红色、生态等旅游精品，促进金门直航湄洲岛朝圣观光常态化，推动台湾妈祖信众朝拜直航包机，建设对台小额贸易市场，创建湄洲岛国家5A级旅游景区。强化服务业市场监管，改善消费环境。积极参与第二次全国经济普查，加强服务业统计监测。

（四）全面深化改革，发展开放型经济

深化重点领域改革。深化乡镇机构、林权制度等农村综合配套改革，稳步推进支农信贷综合试点和农业保险试点工作。完善政府投资体制，强化招投标管理，推广项目法人招标和代建制。积极参与湄洲湾南北岸港口一体化管理体制改革。完善国有资产监管体制，确保国有资产保值增值。充分利用资本市场，指导27家企业改制，力争年内有新的企业上市。推进社会领域改革，重点深化卫生、教育、科技、文化体制改革。

提高招商引资水平。创新利用外资方式，优化利用外资结构，发挥利用外资在推动自主创新、产业升级、区域协调发展等方面的积极作用。积极参加商交会、海交会、“5·18”、“6·18”、“9·8”等招商活动，赴东南亚、广州、南昌等华侨和莆商聚集的地区推介项目，充分利用第五届莆商大会、第四届中国（莆田）医疗器械药品展销会、第十届妈祖文化旅游节、第三届中国（莆田）南少林武术节等招商平台，发挥侨乡优势，推动民资回归，扩大利用外资，引进一批科技含量高、环境污染少、税收贡献大的工业类和服务性项目。

加快外贸转型升级。坚持科技兴贸、以质取胜，促进加工贸易转型升级。加强口岸建设，优化通关环境。顺应出口退税政策和加工贸易政策调整，鼓励企业扩大出口，开拓新兴市场，规避出口风险，实现市场多元化。支持我市企业进入跨国公司采购链，发展服务贸易。扩大先进技术、关键设备和国内短缺能源、原材料进口。

加强莆台港澳合作。举办莆台妈祖文化暨经贸活动，加快莆台农林牧渔业和农产品加工合作，办好台湾农民创业园，建立对台出口商品基地。吸引香港物流、金融、旅游等服务业来莆投资，推动香港服务业与我市制造业对接。加强与澳门合作，吸引葡语系国家及欧盟国家企业来莆投资创业。

（五）建设港口城市，促进区域协调发展

完善城乡规划。认真实施《城乡规划法》，主动衔接国家和省主体功能区规划，完成城市总体规划修编，做好妈祖城及东拓、仙游经济开发区等区域发展规划编制，完成通港大道、莆田火车站、省运会体育场馆周边地区及城市综合商务区详细规划。抓好人防、防震、防洪排涝等一批专项规划。加强村镇建设规划，提高村镇建设水平。

加快建设滨海新区。抓紧实施《妈祖城建设总体规划》，支持中铁集团开发建设妈祖城，加快莆田一中妈祖城校区、新安国际医院和海西度假村、动漫影视基地建设，完善妈祖城核心区路、水、电和污水垃圾处理等基础设施，扎实推进滨海、港里和东秀社区建设。拓展滨海城市空间腹地，建设平海湾路堤工程，筹建妈祖城行政办公中心，促进滨海新城崛起。

推进中心城市建设。提升莆田中心城区、仙游城区城市功能，建设生态宜居城市。围绕省运会和创建国家园林城市目标，做好城港、荔涵、荔园、荔港、仙港、秀屿环城北路等主干道两侧绿化，以及玉湖公园、绶溪公园等园林绿化项目。抓好市政道路、新汽车站、公交场站、自来水、路灯夜景等一批城建项目。继续推进护城河五期、宫口、鲤中、笏石等片区旧城改造。实施木兰溪沿岸总体规划，推进木兰溪防洪二期霞林段和三、四期工程进度，建设商业新区和绿色住宅区，逐步将木兰溪中下游沿岸打造成集商业、居住、休闲于一体的城市繁荣带。加强城市管理，加快城市公共基础设施建设，理顺城市管理执法体制，完善相对集中行政处罚权，大力整治城市环境卫生、市容市貌、交通秩序，治理城市脏乱差现象。

（六）创建生态文明，促进环境友好

加强生态建设。全面推进生态市建设，加快实施一批重大的生态工程，建立一批生态示范区。继续实施沿海防护林、速丰林、城乡绿化一体化与绿色通道工程，建设生态公益林和自然保护区，全市森林覆盖率提高到56.3%。加强农村家园生态环境建设，新推广生态家园3200户，建立以沼气为纽带的生态家园示范村8个，推广“牧-沼-果”生态农业模式3000亩。规范畜禽养殖业，减少环境污染。开展“创绿色家园，建富裕新村”行动，创建全国绿色小康县1个，绿色小康乡（镇）3个，省、市级绿色小康村30个，县级绿色小康村70个。改善木兰溪、萩芦溪等流域水环境，建立重点库区生态保护补偿机制，严格保护东圳水库和金钟水库等饮用水源，保障饮用水安全。加快湄洲岛中国生态文明建设示范基地建设。

加强节能减排。继续创建国家环保模范城市，全面实现市长环保目标责任书年度目标。认真落实节能减排责任制，把完成节能减排目标任务作为检验经济发展成效的重要工作抓紧抓好。滚动实施“1520”节能示范工程，在全市550家企业推广节能新技术、新工艺、新设备，强化20家年耗煤5000吨和年耗电1000万千瓦时以上的重点耗能企业节能管理，抓好高耗能行业设备的更新改造，确保工业生产能耗下降。推进交通和建筑节能，推广新型节能材料。开展污染源普查，严格执行环境影响评价和环保“三同时”制度，完善减排激励约束机制，加强对二氧化硫、化学需氧量重点排放企业监控，严厉查处超标排放企业，确保污染物排放总量不超标。抓紧实施湄洲湾火电厂和其他非电企业脱硫项目，减少二氧化硫的排放量。开工建设市垃圾焚烧发电厂，建成闽中污水处理厂二期工程，建设仙游、秀屿和各工业园区污水处理厂。完善城区污水管网和垃圾转运设施，力争城市生活污水处理率达到70%以上，垃圾处理率达到90%以上。

节约集约用地。修编新一轮土地利用总体规划，合理利用和保护耕地，推进耕地开发和土地整理，确保耕地总量动态平衡。加强项目用地管理和批后监管，清理闲置土地，加强土地市场和矿业权市场管理，规范工业用地“招拍挂”，加强地籍信息化建设，合理开发矿产资源，治理“青山挂白”和水土流失。合理利用海洋资源，节约集约使用海域岸线，发展海洋经济。

（七）建设文化强市，推动文化繁荣

推进文化创新。发展先进文化，建设社会主义核心价值体系，打造莆田文化品牌，提升莆田文化软实力。加快文化设施建设，迁建市艺校、市图书馆，建成莆仙大剧院，筹建市博物馆和福建省莆仙戏剧院。实施文化产业项目带动战略，培育广播影视、出版印刷、戏曲娱乐、传媒广告、网络服务等文化产业，壮大一批文化产业骨干企业。发展哲学社会科学，繁荣新闻出版和文学艺术。继续推进第二届《莆田市志》编撰工作，做好莆仙戏申报“世遗”工作，出版莆仙戏传统戏目丛书，申报国家级历史文化名城，加强文物和知识产权保护。发挥中华妈祖交流协会作用，加强妈祖祖庙、妈祖故里、妈祖阁管理，建设妈祖文化广场、妈祖文化研究院，办好“湄洲妈祖·海峡和平论坛”，筹建妈祖乐府。重视农村文化建设，继续实施农村电影“2131”工程，巩固广播电视“村村通”成果。加强社区、校园、军营、企业文化和外来工文化建设，让人民群众共享文化发展的成果。

发展体育事业。积极筹办第14届省运会，全面动工建设“七馆三场”，整合市体育运动学校等体育资源，培养和引进优秀运动员和教练员，抓好一批优势竞技体育项目，提高竞技体育水平。实施全民健身工程，举办“全民健身与奥运同行”主题活动，开展妇女、老年人和农民运动会等体育竞赛。鼓励外资、民资投资体育产业。

深化精神文明建设。加强公民道德建设，提高市民文明素质。扎实推进未成年人思想道德建设，促进未成年人健康成长。深化政务诚信、商务诚信、社会诚信建设，构建诚信型社会。深入开展群众性精神文明创建活动，加大文明城市、文明县城（区）创建力度，全力迎接全省文明城市、文明县城（区）考评。做好全国、全省精神文明创建等各类先进推荐、评选工作。加强农村精神文明建设，树立农村文明新风。

（八）高度重视民生，促进社会和谐

办好人民满意的教育。促进义务教育均衡发展，大力发展职业教育，加快普及高中教育，提高高等教育质量，重视学前教育，关心特殊教育，发展民办教育、远程教育和继续教育。加强教师队伍建设，提高教师素质。加快市教师进修学院、市特殊教育学校新校区建设。做好莆田学院和湄洲湾职业技术学院迎接教育部评估工作。坚持教育公益性质，规范教育收费，扶持农村偏远地区和海岛教育，健全农村义务教育经费保障机制，逐步化解农村“普九”债务，免除义务教育阶段学杂费，免费为农村义务教育阶段学生提供教科书，补助农村义务教育阶段寄宿生生活费。加大对家庭贫困学生资助力度，保障经济困难家庭、进城务工人员子女接受义务教育。

扩大劳动就业规模。实施积极的就业政策，认真贯彻《劳动合同法》和《就业促进法》，加强高校和职业学校毕业生就业服务，落实就业再就业小额担保贷款政策，积极帮助零就业家庭成员就业，新增城镇就业2.1万人，下岗失业人员再就业2000人。加强对农民实用农业技术和职业技能培训，转移农村劳动力3.5万人。规范和协调劳动关系，及时查处拖欠农民工工资案件，维护劳动者合法权益。

提高医疗卫生保障水平。扩大新型农村合作医疗覆盖面，完善城镇职工基本医疗保险，全面开展城市居民医疗保险工作，开展城镇居民基本医疗保险试点工作，发展城市社区卫生服务，健全城乡三级卫生服务网络，鼓励发展民办医院。加快建设莆田学院附属医院，迁建市皮肤病防治院，建设麻风病村新楼，建成涵江医院门诊大楼，规划建设城厢医院。深入开展“县级以上医疗卫生机构对口帮扶乡(镇)卫生院”和“千名医生帮扶山区乡镇卫生院”活动，实行农村困难家庭医疗救助，为群众提供安全、有效、方便、价廉的基本医疗服务。提高重大疾病、各种传染病预防控制和医疗救治能力。开展爱国卫生运动，发展妇幼卫生事业。

健全社会保障体系。深化企业、机关、事业单位基本养老保险制度改革，探索建立失地、失海农民和农村养老保险制度。扩大各类社会保险覆盖面，严格社会保险基金的征缴和管理。加强低保资金管理，完善城乡居民最低生活保障制度。坚持机关党员干部挂钩孤寡病残户和困难户制度。安排好“五老”人员、“五保户”、下岗失业人员、灾区群众等民众生活。发展残疾人事业。重视老龄工作。发展社会福利、慈善、红十字等公益事业，基本建成市福利院、慈康医院。加快廉租房、经济适用房和“侨居造福工程”建设，逐步解决低收入家庭住房困难。

完善社会管理。坚持计划生育的基本国策，统筹解决人口问题，稳定低生育水平，提高人口素质，优化人口结构。深入开展产品质量和食品药品安全专项整治活动，努力建设消费放心城市。加强信访工作，完善多元化调节衔接机制，抓好社会矛盾纠纷排查调处，努力把矛盾化解在基层和萌芽状态。加强社会治安综合治理，深入开展“平安莆田”创建活动，健全社会治安防控体系，推进公安“三基”工程建设，完善“四队”建设，改革城乡社区警务工作，强化流动人口管理，防范和打击各种违法犯罪行为，特别是“两抢一盗”等侵财性、暴力性犯罪以及“黄赌毒”违法犯罪活动，增强群众安全感。开展“五五”普法教育，加强法律援助。扎实做好国家安全工作。建立健全各类突发性事件的预警机制、应急预案和防灾减灾体系，提高处置社会公共安全危机管理和防灾减灾能力，最大限度地降低社会突发事件和自然灾害造成的损失。落实安全生产责任制，健全安全检查联动机制，有效遏制重特大安全事故。落实拥军优抚安置政策，进一步做好双拥工作，继续创建全国双拥模范城。加强国防动员建设，深入做好民兵预备役、海防、消防和人防工作。

办好惠民实事。着力做好饮水安全村村通工程，技能型人才培训工程，食品药品放心工程，完善基层医疗卫生设施、新型农村合作医疗制度和全面推行科学婚检，农村家园清洁行动，有线数字电视整体转换工程，残疾人康复扶贫助学安居工程，北洋水系二期整治工程，新农村农民文化健身工程，危桥改造工程，城乡公交和邮政配套设施建设工程等11件惠民实事。

三、建设执政为民的服务型政府

各级政府要真诚倾听群众呼声，真实反映群众愿望，真情关心群众疾苦，做到权为民所用、情为民所系、利为民所谋，建设对人民负责、为人民服务、让人民满意的服务型政府。

致力发展。要把发展作为第一要务，不争功，不争利，不争斗，不争论，学习实践科学发展观，争当港城崛起骨干，合心合力攻坚克难，实干实效奋力争先，把心思聚焦在干事业上，把精力集中到抓落实上，解放思想谋划发展，求真务实推动发展，攻坚克难促进发展。坚持把科学发展、和谐发展、全面发展、安全发展贯穿于政府工作的全过程和政府管理的各个环节，加快发展速度，提高发展水平。加强政府公信力建设，强化“一办三中心”职能，努力营造办事效率高、服务质量好、创业成本低、社会环境优的投资发展环境。建立健全促进发展的工作机制，落实好建设海峡西岸经济区和湄洲湾港口城市的各项措施，对认准的事情要扭住不放，决定的工作要一抓到底，全力促进莆田发展。

科学执政。按照经济调节、市场监管、社会管理和公共服务的政府职责，进一步转变政府职能，更加注重运用经济和法律手段调节市场活动，更加注重发挥市场在资源配置中的基础性作用，更加注重依靠政策和服务促进经济发展，把更多的精力放到社会发展和管理上来，把更多的公共资源集中到公共服务上来，把政策的着力点更多地放到解决民生问题上来。完善公共财政制度，发挥财政资金的引导和带动作用，优化财政支出结构，新增财力要更多用于推进重点产业、新农村、社会事业、社会保障和公共安全等方面建设，加强政府专项资金和债务管理，有效防范和化解债务风险。整合国有资产资源，集中力量办大事。深化绩效评估，健全政府激励约束和评价机制。

民主执政。坚持向人大及其常委会报告工作的制度，严格执行人大及其常委会的各项决议，依法接受人大及其常委会的监督，认真办理人大代表的议案和建议、批评、意见。完善与政协的联系和协商制度，认真办理政协提案，充分发挥政协参政议政的职能。认真听取各民主党派、工商联和无党派人士意见，支持工会、共青团、妇联等人民团体依照法律和各自章程开展工作，参与社会管理和公共服务，维护群众合法权益。发挥宗教人士和信教群众在经济社会发展中的积极作用。提高政府决策水平，完善领导、群众、专家相结合的决策机制，完善公示和听证制度，推进科学决策。继续邀请人大代表、政协委员、人民团体和各界人士参加政府常务会。

依法执政。贯彻《全面推进依法行政实施纲要》，推行行政执法责任制，提高行政机关执法水平。强化行政执法监督，做好行政复议和行政诉讼应诉工作。推进政务公开，加强电子政务建设，认真实施政府信息公开条例，充分发挥政府信息对人民群众生产、生活和经济社会活动的服务作用。主动接受新闻媒体的舆论监督，完善办事公开制度和监督机制，提高政府工作透明度，使权力在阳光下运行。深化行政审批制度改革，该放的权力要主动放，该减的程序要果断减，该降的门槛要坚决降，不该收的费用一律不准收，真正履行职能，做到既不越位也不缺位。加强对中介组织的监管。

从严治政。严格执行廉洁从政各项规定，严格落实党风廉政建设责任制，开展政风行风评议，坚决纠正损害群众利益的不正之风。加强机关效能建设，规范公务员从政行为。健全质询、问责、经济责任审计、引咎辞职、罢免等制度，落实行政过错追究制，确保行政权力正确行使。严厉打击以权谋私、权钱交易和商业贿赂行为，严惩贪污腐败分子。对那些不给好处不办事，少给好处慢办事，多给好处乱办事，为民服务态度差、工作不负责任、假公济私的人，不留情面，坚决处理。提倡艰苦奋斗，反对铺张浪费，降低行政成本，少花钱，多办事，花小钱，办大事，建设节约型政府。

各位代表，党的十七大指引我们踏上了跨越发展的新征程，兴化大地焕发着蓬勃发展的盎然生机，莆田的前景十分美好。让我们高举中国特色社会主义伟大旗帜，深入贯彻落实科学发展观，在中共福建省委、省政府和中共莆田市委的坚强领导下，立足新起点，迎接新挑战，创造新业绩，实现新跨越，共同迎接湄洲湾港口城市灿烂辉煌的明天！
